# Zoonotic Threats: The (Re)emergence of Cercarial Dermatitis, Its Dynamics, and Impact in Europe

**DOI:** 10.3390/pathogens13040282

**Published:** 2024-03-26

**Authors:** Maria Teresa Bispo, Manuela Calado, Isabel Larguinho Maurício, Pedro Manuel Ferreira, Silvana Belo

**Affiliations:** Global Health and Tropical Medicine (GHTM), Associate Laboratory in Translation and Innovation Towards Global Health, LA-REAL, Instituto de Higiene e Medicina Tropical, Universidade Nova de Lisboa (UNL), Rua da Junqueira 100, 1349-008 Lisboa, Portugal; mcalado@ihmt.unl.pt (M.C.); isabel.mauricio@ihmt.unl.pt (I.L.M.); pedroferreira@ihmt.unl.pt (P.M.F.)

**Keywords:** zoonotic disease, swimmer’s itch, cercarial dermatitis, *Trichobilharzia*, Europe, One Health

## Abstract

Cercarial dermatitis (CD), or “Swimmer’s itch” as it is also known, is a waterborne illness caused by a blood fluke from the family Schistosomatidae. It occurs when cercariae of trematode species that do not have humans as their definitive host accidentally penetrate human skin (in an aquatic environment) and trigger allergic symptoms at the site of contact. It is an emerging zoonosis that occurs through water and is often overlooked during differential diagnosis. Some of the factors contributing to the emergence of diseases like CD are related to global warming, which brings about climate change, water eutrophication, the colonization of ponds by snails susceptible to the parasite, and sunlight exposure in the summer, associated with migratory bird routes. Therefore, with the increase in tourism, especially at fluvial beaches, it is relevant to analyze the current epidemiological scenario of CD in European countries and the potential regions at risk.

## 1. Introduction

Many water-borne diseases are emerging due to various factors. Among these emerging diseases is cercarial dermatitis (CD), a snail-borne zoonosis that can occur in both fresh and marine waters, resulting from the penetration of trematode cercariae into human skin [1,2]. One factor for this emergence is the increase in leisure and work activities in cercaria-infested freshwater bodies, where intermediate host snail habitats are present [1,3]. Other factors include climate change, which contributes to changes in aquatic bird and freshwater snail populations.

The first scientific reports using the term CD in Europe occurred around the 1930s, shortly after the name was coined in the United States in 1928 [4]. Nevertheless, this disease has acquired the popular name “swimmer’s itch”, reflecting the main symptom and how it is acquired [5]. The parasite invades the dermis, causing severe itching and other allergic reactions, such as oedema and erythema of the affected area [1,6]. CD is caused by Schistosomatidae in general, including the genus *Schistosoma* (the causative agents of schistosomiasis], but a large proportion of the species described in European studies are avian schistosomes [7], reflecting the high prevalence of infection in birds [8], which are able to penetrate human skin instead of their definitive host but do not complete their life cycle.

CD has global distribution, especially due to the migratory patterns of birds, which increases its risk of emergence in areas where no cases have been previously reported [9]. Most reported CD cases, including in Europe, and thus the primary focus of research, relate to freshwater bodies. However, studies conducted in other countries, including the USA, have highlighted cases of CD occurring in seawater [10]. In Africa and Asia, although species of the genus *Schistosoma* are the most studied due to their great impact on human health, other genera of Schistosomatidae have also been reported as responsible for CD cases [11,12]. Furthermore, avian parasite species primarily described in Europe have recently been reported elsewhere, such as Iran [12], with dispersion associated with migratory birds, but most species are reportedly confined to the European continent possibly due to parasite–intermediate host specificity [13]. 

Recently, some European countries have reported an increase in the number of CD cases, with new cases in locations with no previous records of infection [14]. The etiological agents were primarily parasite species of the genus *Trichobilhazia*, which have lymnaeid intermediate hosts [8]. Underreporting is a problem due to possible missed or overlooked diagnoses [7], making it difficult to measure the full economic impact [8], although the tourism industry emerges as a visibly affected sector due to beach closures [15]. 

This review focuses on the current epidemiological pattern of CD in Europe, including its broader impact on European countries, with a brief reference to the CD scenario outside the continent, based on an extensive review of international published papers on this subject.

## 2. Etiologic Agents 

Avian schistosomes are commonly distributed worldwide, and the prevalence of infection in intermediate host snails may vary amongst localities and years, but no correlation was observed with factors such as latitude [8]. They are commonly associated with freshwater habitats such as lakes, ponds, and rivers, where birds and humans come into contact with water [1]. Globally, the main described etiological agents of CD are trematodes of the genus *Trichobilharzia*, but clinical manifestations in humans can be due to other avian schistosomes, such as genera *Dendritobilharzia*, *Gigantobilharzia*, *Allobilharzia*, *Austrobilharzia*, *Anserobilharzia*, *Bilharziella*, *Macrobilharzia*, *Ornithobilharzia*, and *Jilinobilharzia* [1,16]. Although the genus *Schistosoma* can also cause CD in some parts of the world [1], in this review, we will focus on avian schistosomes (mostly of the genus *Trichobilharzia*) as the main agent of CD in Europe.

In Europe, CD primarily occurs in freshwater bodies and arises from species of the genus *Trichobilharzia* [1,17], which rely on freshwater snails as intermediate hosts and birds as definitive hosts [2]. The cercariae of this group had been classified as *Cercaria ocellata* by La Valette (1855), a name that persisted for some time. However, bird parasites originally obtained from a given snail species were not always able to infect other snail species. This observed intermediate host specificity led to the discovery that CD agents belonged to different species, and the name *C. ocellata* was abandoned in Europe [18]. Recognized CD etiological agents on the European continent include the species *Trichobilharzia szidati* [19], *Trichobilharzia regenti* [20], *Trichobilharzia franki* [21], *Trichobilharzia physellae* (Austria) [22], *Trichobilharzia anseri* (Iceland) [23], *Trichobilharzia mergi* (Iceland) [24], and *Trichobilharzia salmanticensis* [25], the latter reported in only a few studies. One characteristic shared among the cercariae of this group is the presence of two pigmented eye spots and the furcocercarial shape (bifurcated tail), which enhance their swimming capacity and rapid tissue penetration [18].

The most prevalent species in Europe (Table 1) have been shown to exhibit specificity with snail hosts, such as *T. szidati* with lymnaeids *Lymnaea stagnalis* and *Radix* sp., *T. franki* with *L. stagnalis* and *Radix* sp., *T. regenti* with *Radix* sp. [1,2,18], and *T. physellae* with the physid *Physa acuta* [1,22]. Additionally, *Bilharziela polonica* demonstrates high specificity with the planorbid *Planorbarius corneus* [22,26]. 

## 3. Intermediate and Definitive Hosts

The most commonly observed intermediate hosts are freshwater snails, such as planorbids, lymnaeids, and physids [7]. *Lymnaea stagnalis* is the most frequently reported intermediate host of avian schistosomes in Europe, with widespread geographical distribution across this continent [60]. Infection of this snail species with *T. szidati* has different effects in juveniles and adults: juvenile forms have their sexual development compromised, while adults increase their oviposition rate, seemingly due to disruption of snail neuroendocrine control [61]. Notably, a single infected *L. stagnalis* can generate CD cases, as it can release over 30,000 cercariae in one day [60]. In Europe, the epidemiology of human CD is significantly influenced by the prevalence of avian schistosomes in freshwater snails, with recorded prevalence rates ranging from 0.05% to over 50% [8].

Parasite and snail host specificity occurs through various mechanisms. Molecules released by snails may function as pheromones, attracting the parasites to suitable host snails [8,62]. In instances where miracidia successfully penetrate the snail’s mucosa, the host’s immunological response is primarily mediated by hemocytes through encapsulation of the parasite [8]. However, the parasite employs various strategies to evade the snail’s immune system, including mimicry or hemocyte activity suppression, thereby ensuring its successful development to the cercarial phase [8,63,64]. 

The definitive hosts of *Trichobilharzia* sp. are waterfowl, primarily Anseriform birds [18,65], of which, in Europe, the most common species are *Anas platyrhynchos* (mallard), *Anas crecca* (common teal), *Anas clypeata* (northern shoveller), *Aythya fuligula* (tufted duck), *Cygnus olor* (mute swan), *Anser anser* (greylag goose), and *Mergus merganser* (goosander). The migratory behaviour of these species facilitates the dissemination of avian schistosomes, notably the *Trichobilharzia* group, along their routes [23,45]. In experimental studies on the development of infection by *T. regenti* in the definitive host (ducks) and in a mouse model [20], it was observed that, after penetrating the skin of ducks and mice (mammal model), this species tends to reside in peripheral nerves, passing through the meninges and potentially causing neural symptoms, including leg paralysis [20,66]. The parasites end up in the nasal cavity, where oviposition occurs, as eggs (mature and immature) as well as miracidia that hatched outside the water were observed in the nasal cavity of the duck model [66].

Among migratory birds, the Egyptian goose has recently adapted to several European countries and it has been found with infections by other platyhelminths, including some trematodes not previously described [67,68]. This bird species is common in certain African countries, such as Rwanda, where it has been described as hosting species such as *Trichobilharzia spinulata* [69,70]. Another Anatid species introduced to Europe is *Oxyura jamaicensis*, originating from the USA and now present in multiple European countries [71,72]. While it remains uncertain whether this bird species can serve as a definitive host for the *Tricobilharzia* group in Europe, the possibility cannot be ruled out, given its susceptibility to other trematodes and the known capacity of *T. regenti* to infect a large number of Anatids [1,73]. Although swimmer’s itch caused by *T. regenti* has not been described so far, the possibility is not excluded, considering that Anatids are already recognized as hosts for digenic trematodes. Adaptation of *T. regenti* to European aquatic environments could expand the pool of definitive hosts and increase the potential for infection of accidental hosts, including humans. This possibility is substantiated by experimental investigations, as migration of the parasite among organs has been observed even in mammalian models, including in the lungs [45].

## 4. Parasite Development, Biology, and Pathology

**Figure 1:**
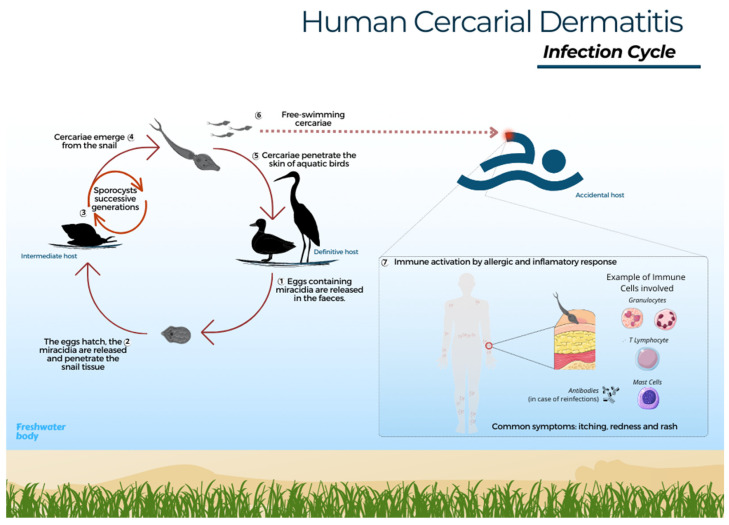
Human cercarial dermatitis – infection cycle. 1- Eggs found in the faeces of infected aquatic birds hatch into miracidia upon contact with water. 2- Miracidia then seek out a specific snail host and penetrate its mucosa. 3- Within the snail, the cycle progresses through the sporocyst phase and subsequent generations; 4- Emergence of infectious cercariae. 5- These cercariae penetrate the skin of the definitive avian host, shedding their tail. Then, schistosomula migrate through blood vessels to various organs, where they develop into adult forms, initiating the sexual phase. 6- Free-swimming cercariae may penetrate human skin, leading to dermatitis. 7- The immune system responds with an allergic and inflammatory reaction, involving the recruitment of neutrophils, mast cells, eosinophils, and T lymphocytes; these cells release cytokines to regulate the inflammatory process and aid in elimination of the parasite.

The parasite life cycle begins when the faeces of infected birds are released into water or near the shore, where the eggs hatch and the miracidia emerge and swim in search of a snail [18]. *Trichobilharzia szidati* miracidia survive approximately 20 h at 20 °C, while species of the genus *Schistosoma* last up to 16 h at 15 °C [19,74]. In general, species of the *Trichobilharzia* group have high specificity toward particular snail species [18]. Miracidia are attracted by glycoproteins present in the specific snail host and ultimately penetrate the cephalopodal region [18,75]. After miracidia penetration, the sporocyst mother, and sometimes the sporocyst daughter, stage develop and eventually produce cercariae [8,76]. Upon release from the snail, the cercarial lifespan depends on water temperature and can last up to a full day [77]. Thus, at the first opportunity to invade any dermal tissue, avian or mammalian, these cercariae begin a mechanical and chemical process of penetration by losing their tail and releasing enzymes to facilitate skin perforation (Figure 1) [13,78].

In birds, schistosomatid larvae can be observed in visceral or nasal locations, depending on the species. In other words, the cercariae develop into schistosomula and migrate through the bloodstream to the organs, and in some species, even to the central nervous system toward the nasal cavity, and after maturing into adults, they begin their reproductive phase [1,79].

When cercariae come into contact with humans, an accidental host, a hypersensitivity reaction occurs, followed by an inflammatory response. Upon reinfection, there may be a subsequent release of antibodies and interleukins to help eliminate the parasite [13,80]. In other words, the immune system responds by activating cells and defence pathways against invasion. Some insights about the infection in mammals have been achieved through experimental animal studies, where it was possible to observe the migration of cercariae to other organs [5]. Experimental studies in mice have shown that the cells involved in the response to, for example, *T. regenti*, are neutrophils, eosinophils, mast cells, and T lymphocytes, which, by releasing inflammatory mediators, cause itching and local tissue damage [17,80]. The immune response modulation aims to eliminate the invasive cercariae, leading to the characteristic symptoms of itching, redness, and rash associated with CD. Penetration by a large number of cercariae may cause increased pruritus and intensify the signs and symptoms of CD [13]. Research thus suggests that, in an accidental host, cercariae are eliminated by the immune system and die in the dermis; however, cases of entry into the blood vessels and migration between organs have been reported in experimental mammal models [17,81]. In the accidental host, the parasite does not differentiate sexually, and the cycle is interrupted.

## 5. Cercarial Dermatitis in Europe: What Do We Know?

**Figure 2:**
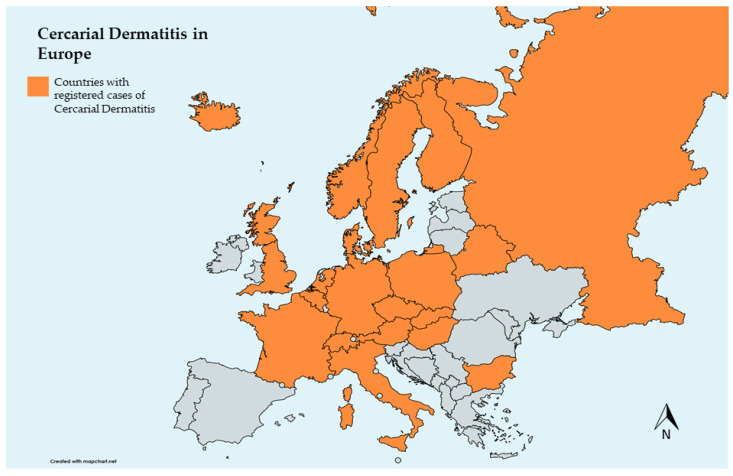
Map of European countries that registered cases of cercarial dermatitis (bicontinental countries were excluded, except for Russia).

The emergence of diseases in Europe has been increasingly discussed due to their public health and economic impact [82], many of which are zoonotic [83]. With significantly higher tourist influx, notably to freshwater beaches, there has been a recent increase in CD case notifications in some European countries, with the majority occurring in the summer (Figure 2) [3,42,84,85]. Considering climate change and schistosomatid trematode developmental characteristics, European countries face a potential risk for CD increase [86]. Similarities between aquatic habitats used for recreation in Europe could facilitate the spread of these parasites, particularly, but not only, between neighboring countries due to the expansion of migratory birds toward northern regions [87]. In particular, rising water temperatures favor the spread of the parasite and consequent infection, whether in the definitive or accidental host [13]. Thus, CD is becoming an emerging, and sometimes reemerging disease that may have a negative impact, especially on tourism, bringing the need for control or mitigation measures. This impact may be underestimated due to undiagnosed cases. Therefore, it is important to encourage clinicians to obtain an inclusive medical history encompassing CD, encouraging its notification, to facilitate investigations in affected regions. The negative effects of CD on human health could be mitigated by enhancing freshwater users’ understanding of the infection and knowledge about risk areas, including water quality, snail presence, and bird populations [1].

Studies conducted in Europe provide insights into the species involved in CD across the continent, encompassing birds, snails, and parasites [46]. Some investigations often employ molecular approaches, revealing similar species in neighbouring countries or even in those not sharing borders [28,88]. In general, the studies have consistently identified the prevalent intermediate hosts, specifically the genera *Radix* and *Lymnaea*, associated with causative agents such as *T. szidati*, *T. franki*, *T. regenti*, *T. physellae* (Austria), *T. anseri* (Iceland), *T. mergi* (Iceland), and *B. polonica*, as the primary contributors to CD in Europe [22,23,24,56,89]. The studies identified were not carried out in all European countries, but mainly in central and northern Europe, indicating significant gaps in the evaluation of the full extent of this zoonosis, maybe due to undiagnosed cases masking the true CD prevalence in other countries, such as Portugal. Research presents challenges, inclusively due to fluctuations in snail populations induced by environmental changes [86,90]; however, further research is crucial, particularly considering that some migratory bird populations are becoming resident in southern regions [91] with favourable climate conditions [92].

Despite the initial awareness of *L. stagnalis* infected with *T. szidati* in northern Polish lakes and subsequent human risk assessment tests in 2004 [93], molecular studies confirmed the presence of both *T. szidati* and *T. regenti* only several years later, from 2018 [54,94]. This finding allowed the characterization of snails (*L. stagnalis*, *Radix balthica*/*labiata*, *Radix auricularia*, and *P. corneus*) infected with digenic larvae (*B. polonica* and *Trichobilharzia* sp.) [89]. In Germany, studies on freshwater areas, such as Lake Baldeney, Ruhr River, and Lake Tunisee, confirmed the presence of *T. franki* in the latter, emphasizing the need for prior knowledge for preventive measures in recreational waters [21,79,95,96]. In the Netherlands, molecular analysis has been employed to detect the presence of *Trichobilharzia* sp. in water at recreational bathing sites, drawing attention to the potential correlation between snail research and environmental investigations [97,98], deepening our understanding of the CD scenario in the country.

Research conducted across European regions has elucidated the substantial role of avian species in CD dissemination. De Gentile et al. [99] already commented on the possibility of CD emergence in several countries. French investigations into bird migration patterns and their interactions with aquatic habitats, particularly at Lake Annecy, have led to various control interventions to mitigate the negative impact on tourism [42,100], such as bird hunting, given the diverse avifauna (migratory and non-migratory), and mechanical snail control [44]. However, other lakes, such as Lake Der-Chantecoq, experienced an exponential increase in the emergence of CD cases within a few years at the beginning of the 21st century [42]. Phylogenetic studies undertaken in France have led to a deeper understanding of parasite haplotype diversity, including in relation to other countries and continents, given the significant number of migratory birds in the region [23,44,46]. In Switzerland and France, Lake Geneva remains a potential source of CD due to its bird population and migratory fluxes, as they straddle the border, with potential infection points distributed throughout its length [58,101,102].

Certain regions have experienced outbreaks of CD, indicating its emerging potential. In Belgium, Lake Eau d’Heure has had numerous reported CD cases caused by *T. franki*, with *R. auricularia* identified as the intermediate host [6]. But recently, in Kampenhout, the increase of CD cases had led to the identification of *T. regenti* in the country, underscoring the importance of monitoring infected snails to avoid outbreaks [30]. The challenge of early *Trichobilharzia* detection in some countries, such as Belgium, may stem from the low abundance of intermediate hosts that may vary seasonally [5]. Slovakia presents a notable example, where evidence of the presence of *T. franki* was only obtained due to outbreak monitoring, specifically at Lake Košice [103]. Until then, studies in the country had been consistently integrated into central European investigations, focusing on the general exploration of trematodes in nations sharing bodies of freshwater [104].

Austria, like several other European countries, has experienced numerous cases of CD, although limited studies had been conducted by the end of the 20th century [105]. Efforts were made to estimate the risk in humans through population questionnaires, providing a valuable means of information dissemination to the community [106]. Molecular studies have since revealed evidence of *T. szidati* and *T. franki* in the country, with *L. stagnalis* and *Radix* sp. serving as intermediate hosts, respectively. Additionally, the presence of *T. physellae* has been confirmed, with the snail *P. acuta* acting as an intermediate host [22,27]. In Hungary, despite some reported cases, the disease has been generally neglected, and only recently have molecular studies been conducted to identify involved species and their distribution [2]. These studies found CD to be caused by *Schistosoma turkestanicum*, with deer as the definitive host, in the Danube River [107]. Furthermore, eggs and adults of *B. polonica*, *Trichobilharzia* sp., and *Dendritobilharzia pulverulenta* were found in organs from bird carcasses, confirming the presence of these trematodes in the country [2,40].

Belarus and Russia have centered their molecular studies around Lake Naroch, located in Belarus [49,74]. The lake, identified as a hotspot with a high number of CD cases, has implemented extensive mollusc elimination measures, particularly targeting *L. stagnalis* [108]. Similarly, Italy has faced outbreaks at various lakes since the initial identification of the infection in the country, leading to the molecular discovery of *T. franki* in *Radix* sp. snails at Lake Vico [52]. The significance of monitoring infection was demonstrated by subsequent outbreaks involving the same trematode species occurring in Lake Albano years later [14].

In countries such as Spain and Portugal, which have water sources that can be considered at risk, studies focused only on human and livestock trematodes [109,110]. Both countries have faced outbreaks of urinary schistosomiasis, Portugal in the 1920s in the Algarve region [111] and Spain, recently, with autochthonous cases in Almería [112]. However, at the end of the 20th century, Simón Vicente and Simón Martin referred to CD cases in Salamanca caused by *T. salmanticensis* [25]. Soldanová et al. [5] points out that the greater number of studies in some countries compared to others may be because their research centers are more focused on CD, which would explain why countries with the same characteristics have few or no studies on this subject.

## 6. An Overview of CD Outside Europe

Countries outside Europe may exhibit similar CD agents and challenges but also differences, as follows. Both the USA and Canada use the cercariometry approach for detection of species involved in CD [113,114], which is not a tool commonly used in Europe. These countries also feature the presence of *Lymnaea* sp., but, in contrast, *Physa* sp. is also an important intermediate host, including *T. physellae* [35,115]. Canada faces challenges with neglected diagnoses of CD; however, people actively participate in citizen science studies, aiding in understanding and mapping the disease’s distribution, as well as predicting its prevalence [116]. In South America, where CD cases are relatively underreported, molecular methodologies are the predominant detection standard [117]. This is exemplified by Ebbs et al. [88], who, in studies of ducks of the genus *Anas* migrating from South Africa, New Zealand, and Argentina to North America, observed a considerable infection rate with *Trichobilharzia querquedulae* and showed that strains were not geographically restricted, punctuating its widespread dissemination. The importance of monitoring avian migratory routes was also confirmed by Ashrafi et al. [12], who documented the emergence of *T. franki* in Iran and molecularly matched the parasite genotype thus far previously exclusive to Europe. As in Europe, various studies have reinforced the need to understand the role of freshwater snails in the development of fluke diseases, such as CD, and devise measures to control the disease without adversely affecting fauna. Climatic factors have also emerged as a major concern, directly and indirectly influencing pathogens and hosts, thereby impacting the prevalence and distribution of CD [92].

## 7. Concluding Remarks

The economic impacts attributed to CD in Europe predominantly affect the tourism sector, particularly with the growing number of individuals engaging in recreational water activities [42,79]. Depending on the level of cercaria infestation in water bodies, certain fluvial beaches may temporarily close as a protective measure until conditions allow for their reopening [89,103]. It is essential to consider the potential occupational risks associated with CD, particularly associated with farming, notably rice fields, where intermediate and vertebrate hosts are present. These farmer workers can experience prolonged and direct exposure to parasites over consecutive hours and days, increasing the likelihood of work absenteeism due to allergic manifestations [1]. This scenario reflects situations observed in Asian countries where CD endemicity is primarily attributed to continuous exposure among farmer workers [11,118]. In response to outbreaks, countries are conducting investigations to assess the CD situation, implementing measures that range from questionnaire-based surveys [106] to cohort follow-up [102], and exploring the potential use of commercially available skin creams as protection for bathers, fishers, and workers [119].

CD is an emerging and re-emerging zoonotic disease in many countries, and in the current context of climate change and global warming, the study of this disease becomes even more relevant. Preventive and control measures should aim to sensitize and educate the population about waterborne diseases without inducing panic to the extent of interrupting water use. Tourism and rice culture, significant regional economic sectors, underscore the importance of maintaining healthy environments by demonstrating concern for both human and animal health through monitoring natural waterbodies. Enhanced collaboration between European countries and institutions investing in knowledge and CD control measures is advisable. In essence, a One Health approach is crucial, whereby molecular detection, characterization, and potential preventive measures can yield more accurate prevalence figures and facilitate more effective interventions.

## Figures and Tables

**Table 1:** Molecular sequences of some schistosomatids that can cause cercarial dermatitis deposited in the GenBank (Europe).

Country	Locality	Stage	Species	Accession Number	Reference
Austria	Reichersberger Au	cercaria	*Trichobilharzia franki*	MT763194-98	Reier et al., 2020 [27]
	Lake Pleschinger See	cercaria	*Trichobilharzia physellae*	OL434662-65	Helmer et al., 2021 [22]
Belarus	Lakes Naroch and Polonevichi	cercaria	*Trichobilharzia szidati*	GU350726; HM001253; HM001260-61	Rizevsky et al., 2011 [26]
		cercaria	*Trichobilharzia franki*	HM001254; HM001256	
		cercaria	*Trichobilharzia* sp.	HM001257-59	
		cercaria	*Bilharziella polonica*	HM001255; HM001262	
	Lake Naroch	sporocyst/ cercaria	*Trichobilharzia szidati*	KP889985-KP890002	Semyenova et al., 2015 [28]
	Lakes Great Shwakshty and Naroch	cercaria	*Trichobilharzia szidati*	MT112075-106; MT708486-99	Chrisanfova et al., 2021 [29]
Belgium	Lake Eau d’Heure	cercaria	*Trichobilharzia franki*	KX034088	Caron et al., 2017 [6]
	Kampenhout	cercaria	*Trichobilharzia regenti*	PP232105	Schols et al., 2024 [30]
Czech		cercaria	*Trichobilharzia franki*	AF356845	Dvořák et al., 2002 [31]
		cercaria	*Trichobilharzia regenti*	AF263829	
		cercaria	*Trichobilharzia szidati*	AF263828	
			*Trichobilharzia regenti*	AY157190; AY157218; AY157244	Lockyer et al., 2003 [32]
			*Trichobilharzia szidati*	AY157191; AY157219; AY157245	
		cercaria	*Trichobilharzia franki*	AY713969; AY713973	Rudolfova et al., 2005 [33]
		cercaria	*Trichobilarzia szidati*	AY713961; AY713968; AY713972	
		cercaria	Avian schistosomatid sp.	AY713963; AY713969	
	Tovačov	egg/ miracidium	*Trichobilharzia regenti*	EF094538; EF094540	Rudolfova et al., 2007 [34]
		fluke	*Trichobilharzia szidati*	EF094541	
		fluke	*Bilharziella polonica*	EF094539	
		cercaria	*Trichobilharzia franki*	FJ174530	Brant & Loker, 2009 [35]
		cercaria	*Trichobilharzia szidati*	GU233735-36 *	Rizevsky et al. 2011 [26]
		cercaria	*Trichobilharzia szidati*	GU233739 *	Aldhoun et al., 2012 [36]
		egg	*Trichobilharzia regenti*	GU233740 *	
	Novozámecký, Litovický, and Dolní Svitavský Ponds, Modřany and Loužek	sporocyst/cercaria/fluke	Avian schistosomatid sp.	FJ786027-30; JF734335; JF694008	Aldhoun et al., 2012 [36]
Denmark	Copenhagen (North)	cercaria	*Trichobilharzia franki*	KP271013	Christiansen et al., 2016 [37]
		cercaria	*Trichobilharzia szidati*	KP271014	
		cercaria	*Trichobilharzia regenti*	KP271015	
	Zealand, Jutland, and Funen	cercaria	*Trichobilharzia* sp. (*Trichobilharzia anseri*)	FJ469784-85; FJ469791	Al-Jubury et al., 2021 [38]
		cercaria	*Trichobilharzia anseri*	MW538530; MW482445	
		cercaria	*Trichobilharzia franki*	MW538531; MW482439-41; MW482443-44; MW482446	
		fluke/ cercaria	*Trichobilharzia szidati*	MW482436-37; MW482447-49	
		fluke	*Trichobilharzia regenti*	MW482450	
England	Tundry Pond (Hampshire)	cercaria	*Trichobilharzia franki*	KJ775865-69	Lawton et al., 2014 [39]
	Knowsley Safari (Prescot)	cercaria	*Trichobilharzia* sp.	ON987329-30	Juhász et al., 2022 [40]
		cercaria	*Trichobilharzia anseri*	ON987331	
		cercaria	*Bilharziella polonica*	ON987332-34	
Finland	Lakes Vuojärvi, Peurunkajärvi, and Konnevesi	cercaria	*Trichobilharzia szidati*	FJ609409-10	Aldhoun et al., 2009 [41]
		cercaria	*Trichobilharzia franki*	FJ609411	
		cercaria	Avian schistosomatid sp.	FJ609412-14	
France	Marne and Lake Der-Chantecoq	cercaria	*Trichobilharzia szidati*	AY795570-71	Ferté et al., 2005 [42]
		cercaria	*Trichobilharzia franki*	AY795572-73	
	Champagne region	fluke	*Bilharziella polonica*	DQ813437-42	Bayssade-Dufour et al., 2006 [43]
		fluke	*Dendritobilharzia pulverulenta*	DQ813443	
	Lake Annecy	cercaria/egg/ miracidium	*Trichobilharzia regenti*	EU413960; EU413967-70; EU413977-79	Jouet et al., 2008 [44]
		cercaria	*Trichobilharzia* sp.	EU413961; EU413964; EU413970	
		cercaria	*Trichobilharzia franki*	EU413962-63; EU413965-66; EU413971-76	
	Lakes Der-Chantecoq and Annecy, Vanault les Dames, Beauvais, and Forêt d’Orient	fluke/egg	*Trichobilharzia franki*	FJ793813-22; FJ793874-83	Jouet et al., 2009 [45]
		fluke/egg	*Trichobilharzia regenti*	FJ793823-49; FJ793884-95	
		fluke/egg	*Trichobilharzia szidati*	FJ793896-97	
		fluke/egg	*Bilharziella polonica*	FJ793850-57; FJ793898-99; FJ793900-07	
		fluke/egg	Schistosomatidae sp.	FJ793858-73; FJ793908-22	
	Lakes Der-Chantecoq and Annecy, and Beauvais	cercaria/fluke/egg	*Trichobilharzia regenti*	HM439484; HM439487; HM439494-99; HM439500-02	Jouet et al., 2010 [46]
		egg	*Trichobilharzia* sp.	HM439493; HM439505	
	Lakes Der-Chantecoq and Annecy, Beauvais, and Strasbourg	cercaria	*Trichobilharzia franki*	HM131131-41; HM131158-67; HM131176-84; HM13197-99; HM131200-02	Jouet et al., 2010 [47]
		cercaria	*Trichobilharzia* sp.	HM131156-57; HM131192-96; HM131203-05	
	Lake Annecy	cercaria	*Trichobilharzia mergi*	JX456170	Kolarová et al., 2013 [24]
	Lakes Der-Chantecoq and Annecy, and Beauvais	fluke/egg	*Trichobilharzia anseri*	KP901355-56; KP901369; KP901376-79; KP901382-85	Jouet et al., 2015 [23]
Germany	Erlangen	cercaria	*Trichobilharzia occelata*	AF442689	Helter et al., 2002 [48]
		cercaria	*Trichobilharzia regenti*	AF442688	
			*Trichobilharzia occelata*	AY157189; AY157217; AY157243	Lockyer et al., 2003 [32]
		cercaria	*Trichobilharzia szidati*	AY713971	Rudolfova et al., 2005 [33]
		cercaria	*Trichobilharzia franki*	FJ711767-68	Brant & Loker, 2009 [35]
Hungary		fluke	*Orientobilharzia turkestanicum*	EU702749	Majoros et al., 2010 [49]
	Eger, Hortobágy, and Gyulaj	cercaria	*Trichobilharzia franki*	MZ560932-4; MZ562961-63; MZ562965-66	Juhász et al., 2022 [2]
		cercaria	*Bilharziella polonica*	MZ562959-60; MZ562964	
Iceland		fluke	*Allobilharzia visceralis*	DQ067561	Kolarova et al., 2006 [50]
	Reykjavík, Hrísatjorn, Osland, Botnsvatn, Mývatn, Víkingavatn, and Landmannalaugar	cercaria	*Trichobilharzia* sp.	FJ469784-99; FJ469803-04; FJ469807	Aldhoun et al., 2009 [51]
		cercaria	*Trichobilharzia franki*	FJ469805; FJ469806; FJ469808-12; FJ469816-17; FJ469819-21	
		cercaria	Avian schistosomatid sp.	FJ469813; FJ469815; FJ469818; FJ469822	
	Landmannalaugar and Reykjavik	fluke/egg	*Trichobilharzia regenti*	HM439484-86; HM439488-92; HM439503-04	Jouet et al., 2010 [46]
	Botnsvatn, Helgavogur-Myvatn, and Raudavatn	cercaria	*Trichobilharzia* sp.	HM131142-55; HM131168-75; HM131185-91	Jouet et al., 2010 [47]
	Botnsvatn	fluke/egg	*Trichobilharzia mergi*	JX456151-69; JX456170-72	Kolarova et al., 2013 [24]
		cercaria/fluke/egg	*Trichobilharzia anseri*	KP901348-54; KP901357-68; KP901370-75; KP901380-81	Jouet et al., 2015 [23]
Italy	Lake Vico	cercaria	*Trichobilharzia franki*	HM596077	Cipriani et al., 2011 [52]
	Lake Albano	cercaria	*Trichobilharzia franki*	MK053632; MK046867	De Liberato et al., 2019 [14]
Netherlands		cercaria	*Trichobilharzia szidati*	AY713970	Rudolfova et al., 2005 [33]
Norway	Lake Takvatn	cercaria	*Trichobilharzia franki*	KY513270-75	Soldanová et al., 2017 [53]
Poland		cercaria	*Trichobilharzia szidati*	AY713965	Rudolfova et al., 2005 [33]
		cercaria	*Trichobilharzia franki*	AY713964; AY713966	
	Gdansk	egg	*Trichobilharzia szidati*	EF094530; EF094536	Rudolfova et al., 2007 [34]
		egg	*Trichobilharzia regenti*	EF094533-35; EF094537	
		egg	*Trichobilharzia* sp.	EF094531-32	
		cercaria	*Trichobilharzia szidati*	MH190225-28	Marszewska et al., 2018 [54]
		cercaria	*Trichobilharzia regenti*	MH190224	
	Lake Drawsko (West Pomerania Voivodeship)	cercaria	*Trichobilharzia szidati*	MT785880-82	Stanicka et al., 2021 [55]
Russia		cercaria	*Trichobilharzia franki*	GU980749-50	Korsunenko et al., 2010 [56]
		cercaria	*Trichobilharzia szidati*	GU980751-53	
		cercaria	*Trichobilharzia regenti*	GU980754-55	
	Kargat River, Altufyevo and Olympiyskaya derevnya Ponds	cercariae	*Trichobilharzia szidati*	HM016851-57; JF838197-99; JF8381200-03	Korsunenko et al., 2012 [57]
	Moscow ponds and Lake Onega	sporocyst/ cercaria	*Trichobilharzia szidati*	KP890003-21	Semyenova et al., 2015 [28]
Switzerland	Lake Geneva	cercaria	*Trichobilharzia franki*	AJ312041-46	Picard & Jousson, 2001 [58]
		cercaria/fluke	*Trichobilharzia regenti*	AJ312047-49	
Ukraine			*Bilharziella polonica*	AY157186; AY157214; AY157240	Lockyer et al., 2003 [32]
Others (collaborations)		cercaria	*Trichobilharzia regenti*	DQ859919	Webster et al., 2007 [59]

* unpublished.
